# Assessment of Water Resources Carrying Risk and the Coping Behaviors of the Government and the Public

**DOI:** 10.3390/ijerph18147693

**Published:** 2021-07-20

**Authors:** Ning Zhang, Zichen Wang, Lan Zhang, Xiao Yang

**Affiliations:** School of Management, Hangzhou Dianzi University, Hangzhou 310018, China; zhedazhangning@126.com (N.Z.); zhanglan980228@hdu.edu.cn (L.Z.); krisyang@hdu.edu.cn (X.Y.)

**Keywords:** water resources carrying risk, vulnerability of disaster-bearers, hazard of disaster-causing factors, coping behaviors

## Abstract

The carrying capacity of water resources is of great significance to economic and social development, eco-environmental protection, and public health. The per capita water resources in Zhejiang Province is only 2280.8 m^3^, which is more likely to cause the risk of water resources carrying capacity in the case of water shortage. Therefore, this paper applies Analytic Hierarchy Process-Fuzzy Comprehensive Evaluation and Entropy-Principal Component Analysis to evaluate the vulnerability of disaster-bearers and the risk of disaster-causing factors; it comprehensively evaluates the risk of water resources carrying capacity in Zhejiang Province by constructing risk matrix and ranking scores. The specific results are as follows: According to the comprehensive evaluation of the vulnerability of disaster-bearers in Zhejiang Province from the three aspects of supporting force, regulating force, and pressure, the overall performance was good. In particular, the role of supporting force is the most obvious. In the risk of disaster factors, it was found that industrial structure, climate change, water use efficiency, and population structure have great influence, showing that southern Zhejiang is at a greater risk than northern Zhejiang, and western Zhejiang is at a greater risk than eastern Zhejiang, but the overall score gap is not large. Combining the two results, the order of water resources carrying risk in Zhejiang Province from low to high was Hangzhou, Ningbo, Shaoxing, Jiaxing, Huzhou, Jinhua, Quzhou, Wenzhou, Lishui, Taizhou, and Zhoushan. Finally, according to the development planning of different cities, the coping behaviors of the government and the public regarding water resources carrying risk are put forward.

## 1. Introduction

The epidemic situation of COVID-19 swept across the world in 2020. In recent years, the number of occurrences and losses caused by both public health events and natural disasters is on the rise [1]. Therefore, the research on public health events, risk assessment of natural disasters, risk management, and other related fields is becoming increasingly intense.** China is one of the countries most affected by natural disasters in the world. In order to alleviate the possible impact of disasters and seek the harmonious development of man and nature, it is imperative to carry out natural disaster risk research in China. In 1981, Timmerman formally put forward the concept of vulnerability and applied it to disaster risk assessment [2].** Since then, in the international mainstream research on natural disasters and risks, some scholars have been keen to carry out disaster risk from three aspects: the hazards of disaster-causing factors, the exposure to the disaster-prone environment, and the vulnerability of the disaster-bearers. Additionally, in some studies, exposure to the disaster-prone environment and the vulnerability of the disaster-bearers are summarized as the vulnerability of the disaster-bearing body, and it is pointed out that risk assessment is the basis of risk analysis.** As a special natural resource, the lack of effective safety management of water resources leads to many problems, such as flood, drought, water pollution, and water shortage [3].** Furthermore, water resources security risk assessment is an important basis for water resources risk management. China is a water-deficient country, so it is of certain significance to assess the disaster risk caused by water security.

On this basis, scholars have conducted a risk assessment on floods, droughts, water pollution, and water shortage, and have carried out an in-depth study on the factors affecting disaster risk.** Most scholars have carried out research on the hazards and vulnerability caused by disasters. Yu pointed out that vulnerability can be defined as the ability of a region to respond to and resist the effects of natural disasters, while risk can be defined as the possibility of natural or man-made physical events, which can show the occurrence of disaster risks in different ways [4]. As far as flood disasters are concerned, Lian took rainfall and tide level as the disaster-causing factors to evaluate the flood and waterlogging risk of coastal cities and found that rainstorms are the main disaster-causing factor in inland areas, and high tide level is the main disaster-causing factor in island areas [5]. Wang assessed the risk of agricultural flooding and waterlogging disasters in Jilin Province and constructed a rainstorm flood risk assessment index system using four aspects: the harmfulness of disaster-causing factors, the sensitivity of disaster-prone environment, the vulnerability of disaster-affected subjects, and the ability of disaster prevention and reduction; it was also pointed out that extreme precipitation events were the main cause of flood disaster [6].** Bouaakkaz assessed the flood disaster in Susi Basin and found that population size, land abuse, overdevelopment, and other factors rapidly aggravated the vulnerability and susceptibility of flood disasters in this area [7]. Lv constructed a comprehensive evaluation index system of urban flood-bearing risk based on the vulnerability of flood-bearing capacity and the vulnerability of disaster prevention and mitigation capacity to study the flood-bearing risk of Zhengzhou and considered that the rapid development of urbanization is the main reason for the increased risk of urban flood and waterlogging disasters [8].** Agrawal studied the relationship between flood risk and resilience in terms of exposure, susceptibility, and lack of coping capacity [9].** Chen studied the mountain torrents in the Guanshan River Basin and found that with the development of the economy and the migration of population, the risk of mountain torrents is increasing [10].** Based on the conclusions drawn by most scholars, it can be found that the influencing factors that cause or destroy the vulnerability of flooding and waterlogging mainly include geography, nature, society, and human behavior, among which human behavior has a greater influence, and the influence caused by a combination of many factors is more serious. For drought disasters, many scholars also assess the risk of drought disasters in terms of hazards, exposure, and vulnerability from different angles.** Kim used hydrometeorological and socio-economic data to assess the risk and vulnerability of drought and pointed out that there are both high risk and high vulnerability in high-risk areas [11].** According to the relationship between water use and supply, Wen constructed a set of assessment methods for drought and water shortage risk from the three aspects of the disaster, exposure, and vulnerability, indicating that drought conditions will put additional pressure on the water supply system [12]. Ali believed that drought risk refers to potential disaster losses caused by drought events, which was often described as a function of vulnerability, harmfulness, and exposure, and assessed Africa at the national level, pointing out that controlling population growth has been found to be essential for mitigating drought risk in Africa (or even more effective than mitigating climate change) because it improved socio-economic vulnerability and reduced potential drought risk [13].** In summary, it can be seen that risk assessment from two aspects of risk and vulnerability is a common starting point, which has a longer history of use in the risk research of flood, drought, and other disasters in the field of water resources. Therefore, it can be applied to the risk study of water resources carrying capacity.

Water resources carrying risk is the concrete application of disaster risk theory in the field of water resource carrying capacity [14]. The carrying capacity of water resources was first put forward in the study of the development, utilization, and strategy of water resources in China at the end of the 1980s. Jia defined the carrying capacity of water resources as the maximum supporting capacity of local water resources to the economic development and maintenance of a good ecological environment in a region or river basin under specific development stages and development models [15]. It covered all aspects such as economy, society, resources, and ecological environment [16].** In recent years, many scholars had studied the relationship between water resources carrying capacity and water resources shortage risk [17,18], water resources ecological risk [19,20], water resources security risk [21,22], water resources system risk [23,24], and so on, showing that water resource carrying capacity is closely related to water resources risk. However, as a complex system, the carrying capacity of water resources has the possibility of risk generation. Therefore, based on the theory of disaster risk and the theory of carrying capacity of resources and environment, this paper evaluates the risk of carrying capacity of water resources from the hazards of disaster-causing factors and the vulnerability of disaster-bearers.

The innovation of this paper is that the research on water resources carrying risk in China is still in its infancy, and there is no empirical research on it on the basis of theoretical research; secondly, this paper evaluates the vulnerability of disaster-bearers and the hazards of disaster-causing factors, and then comprehensively obtains the specific situation of water resources carrying risk in Zhejiang Province. The following chapters are as follows: Section 1 is a research design, including concept explanation, index setting, and model construction; Section 2 is a specific empirical analysis of the vulnerability of disaster-bearers and hazards of disaster-causing factors; Section 3 includes conclusions, recommendations, and deficiencies.

## 2. Research Design

**Figure 1:**
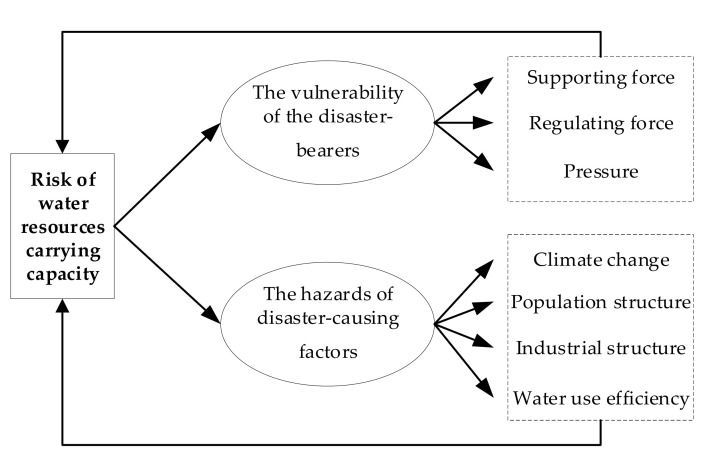
Risk of water resources carrying capacity model.

Long defined the risk of water resources carrying capacity as the probability of water resources overloading events under various uncertain situations, and considered that the risk of water resources carrying capacity is closely related to and complementary to the traditional evaluation of water resources carrying capacity, and the former is the extension of the latter, the latter is the basis of the former [14].** From the point of view of disaster risk assessment, the constituent elements of risk mainly include the disaster-causing factors and the disaster-bearers; the regional disaster risk level is affected by the vulnerability of the disaster-bearers and the hazards of disaster-causing factors. According to this, Long summed up the theoretical model of water resources carrying risk, as shown in Figure 1.

### 2.1. The Vulnerability of the Disaster-Bearers

#### 2.1.1. Index Setting

As the research on water resources carrying capacity has become mature in China, and as the basis of water resources carrying risk, academia has a set of relatively rigorous evaluation systems. Therefore, according to the existing mature research, we can summarize a disaster-bearing subject vulnerability index system and its corresponding classification criteria, as shown in Table 1.

#### 2.1.2. Model Building

As the vulnerability of disaster-bearers has the characteristics of uncertainty and ambiguity, on the basis of referring to the research of other scholars, this paper uses Analytic Hierarchy Process- Fuzzy Comprehensive Evaluation model to evaluate the vulnerability of disaster-bearers regarding water resource carrying risk in Zhejiang Province so as to effectively reflect the results. The Fuzzy Comprehensive Evaluation model is constructed as follows:

Suppose that the two finite field theories are:

$$U=\left\{U_{1},U_{2},\cdots ,U_{n}\right\},V=\left\{V_{1},V_{2},\cdots ,V_{n}\right\}$$

*U* represents a set of factors that affect the evaluation object, and *V* represents a set of comments. *B = A × E* represents fuzzy comprehensive evaluation, *A* represents fuzzy subset on *U*, $A=\left\{a_{1},a_{2},\cdots ,a_{n}\right\},0\leq a_{i}\leq 1$, *a_i_* represents the membership degree of *U_i_* to *A*. It not only represents the role of a single factor *U_i_* in the evaluation factor but also represents the ability of *U_i_* evaluation grade to some extent. $B=\left\{b_{1},b_{2},\cdots ,b_{n}\right\},0\leq b_{j}\leq 1$, *B* is the result of the evaluation and is a fuzzy subset on *V_j_*. The membership degree of grade *V_j_* to the fuzzy subset *B* obtained by comprehensive evaluation is *b_j_*. The evaluation matrix *R* is:

$$R=\left|\begin{array}{cccc} r_{11} & r_{12} & \cdots & r_{1n}\\ r_{21} & r_{22} & \cdots & r_{2n}\\ \vdots & \vdots & \ddots & \vdots \\ r_{n1} & r_{n2} & \cdots & r_{nn} \end{array}\right|$$

where *r_ij_* represents the membership degree of *U_i_* to grade *V_j_*, $r_{i}=\left\{r_{i1},r_{i2},\cdots ,r_{in}\right\}$ indicates the results of the single factor evaluation of the *i* factor *U_i_*. The comprehensive evaluation is mainly based on the value of the quantitative evaluation set and the assignment of each grade membership degree in matrix *B*, and is calculated according to the following formula:

(1) $$a=\frac{\sum_{i=1}^{3}b_{i}^{k}a_{i}}{\sum_{i=1}^{3}b_{i}^{k}}$$

In Formula (1), the *a* value represents the comprehensive score of the vulnerability of the disaster-bearers based on the fuzzy comprehensive evaluation result matrix *B*.** The *r_ij_*, in the evaluation matrix *R* can be compared and analyzed by the actual value of the evaluation factors and the grading index of each evaluation factor, and then the results can be calculated. For grade II, that is, the middle part, the membership degree of the middle point is 1, the membership degree of both edges is 0.5, and the membership degree of the middle point to both sides decreases linearly; for grade I and grade III, the farther away from the critical value, the greater the membership degree of both sides. On the critical value, the membership degree of both sides is 0.5. In order to make the membership function transition smoothly between different levels, it is necessary to fuzzify the membership function. Here, I, II, and III are defined as *V_1_*, *V_2,_* and *V_3_* respectively.

According to the above assumptions, the calculation formulas of membership functions of each evaluation grade are established. The critical value between grade I and II is expressed by *k_1_*, the critical value between grade II and III is expressed by *K_3_*, and the midpoint value of grade II is expressed by *k_2_*, and *k_2_* = (*k_1_+k_3_*)/2. The formula for calculating the membership degree of each evaluation factor to the grade is as follows:

(2) $$U_{V_{1}}=\{\begin{array}{l} 0.5\left(1+\frac{u_{i}-k_{1}}{u_{i}-k_{2}}\right),u_{i}<k_{1}\\ 0.5\left(1-\frac{k_{1}-u_{i}}{k_{1}-k_{2}}\right),k_{1}<u_{i}<k_{2}\\ 0,u_{i}>k_{2} \end{array},U_{V_{2}}=\{\begin{array}{l} 0.5\left(1-\frac{u_{i}-k_{1}}{u_{i}-k_{2}}\right),u_{i}<k_{1}\\ 0.5\left(1+\frac{k_{1}-u_{i}}{k_{1}-k_{2}}\right),k_{1}<u_{i}<k_{2}\\ 0.5\left(1+\frac{u_{i}-k_{3}}{k_{2}-k_{3}}\right),k_{1}<u_{i}<k_{2}\\ 0.5\left(1-\frac{k_{3}-u_{i}}{k_{2}-u_{i}}\right),u_{i}>k_{3} \end{array},U_{V_{3}}=\{\begin{array}{l} 0,u_{i}>k_{3}\\ 0.5\left(1-\frac{u_{i}-k_{3}}{k_{2}-k_{3}}\right),k_{2}<u_{i}<k_{3}\\ 0.5\left(1+\frac{k_{3}-u_{i}}{k_{2}-u_{i}}\right),u_{i}<k_{2} \end{array}$$

Thus, the water resources disaster-bearers matrix *R_i_*, in *i* City, Zhejiang Province is obtained, and the evaluation value is determined by multiplying the weight matrix obtained by the analytic hierarchy process with the water resources disaster body matrix Ri. Finally, the risk matrix among the first-level indexes is constructed according to the evaluation results, and the vulnerability risk matrix of the final disaster-bearers is formed by pairwise combination.

#### 2.1.3. The Concept and Composition of the Risk Matrix

At the end of the last century, the concept of a risk matrix was first put forward in the United States, and it was initially used to solve risk management problems in the chemical industry and various projects.** Subsequently, the risk matrix became widely used in various fields because of its simplicity and intuitive nature. In the risk matrix, the risk criteria are often evaluated by consequences and possibility [25].** When using the risk matrix, deviations can occur when people enter the data, which may lead to different results in the assessment of the same risk [26], and the risk preference cannot be well embedded in the risk matrix [27].** Baybutt believed that in risk management, many scholars use a risk matrix to rate the risk of dangerous scenarios to determine the necessity of risk reduction [28].** An evaluation risk matrix of water resource carrying capacity has been put forward by Jin, in which the pressure, supporting force, and regulating force are regarded as the risk factors affecting water resources carrying capacity [29].

In this paper, based on the risk matrix of water resources carrying capacity, the vulnerability risk matrix of disaster-bearers is further improved. As shown by Table 2, the evaluation grades of row coordinates and column coordinates include grade III, grade II, and grade I, which means vulnerable, a little vulnerable, and not vulnerable, respectively. In the disaster-bearers supporting-regulating force composite matrix, the row coordinates represent the disaster-bearers regulating the force grade; then, the regulating force increases step by step from the bottom to top, while the column coordinates represent the disaster supporting force grade; then, the supporting force increases step by step from right to left, so the other two composite matrices can be inferred. Due to the different evaluation areas and the change of evaluation criteria, we can fully consider the role of disaster bearing and the control of water resources to reasonably select the synthesis rules of the three risk matrices to obtain the vulnerability evaluation level of disaster-bearing subjects. In this paper, the synthesis rules of the risk matrix are determined comprehensively with reference to [29].

The composition rules of each risk matrix are as follows: for the disaster-bearers supporting-regulating force composite matrix, when the supporting force is a grade I or II, because of the high weight of the supporting force, the composite grade is the supporting grade. When the disaster-bearers support force is grade III, the disaster-bearers regulating force can play a better regulating role only when it is in grade I, so the composite grade can be taken as grade II, and, under other conditions, the composite grade is the supporting force grade. For the disaster-bearers pressure-regulating force synthetic matrix, when the pressure is a grade I or II, the composite grade is the pressure grade because of the high weight of the pressure; when the pressure is a grade III, at this time, the regulating force can be adjusted when the regulating force is at grade I or II, so the composite grade can be taken as grade II, and under other conditions, the composite grade is the grade of supporting force. For the composite matrix of the disaster-bearers supporting-regulating force and the disaster-bearers pressure-regulating force, when the grade of supporting-regulating force is the same as that of pressure-regulating force, the composite grade is the same grade for both of them. When the grade of the supporting-regulating force is higher than that of the pressure-regulating force, and there is only a difference of one degree, the composite grade is the disaster supporting-regulating force grade. If there is a difference between the two grades, the composite grade will be reduced to a grade II due to the influence of pressure. When the pressure-regulating force grade is II and the supporting-regulating force grade is I, or the pressure-regulating force grade is III and the supporting-regulating force grade is II, because of the higher weight of supporting-regulating force, the composite grade is the pressure-regulating force grade. When the pressure-regulating force grade is grade III and the supporting-regulating force grade is a grade I, the pressure-regulating force plays a certain regulatory role, so the composite grade is grade II.

### 2.2. Hazard of Disaster-Causing Factors

#### 2.2.1. Index Setting

According to the reference [14], the risk assessment index system of disaster-causing factors is established in this paper, as shown in Table 3.

#### 2.2.2. Model Building

1. Index Data Processing

Set data matrix $X={\left(X_{ij}\right)}_{n\times m}$, where *X_ij_* is the value of the *j* index of the *i* subsystem, *i* = 1, 2, …, *n*, specifically:

(3) $$X=\left(\begin{array}{ccc} X_{11} & \cdots & X_{1m}\\ \vdots & \ddots & \vdots \\ X_{n1} & \cdots & X_{nm} \end{array}\right)$$

Because the nature of each index is not the same, it is necessary to standardize the original data and eliminate the dimensions to make them comparable, so Z-score standardization is used in the principal component analysis. Standardize the data in the *X* matrix by Z-score, and the formula is $Z=\left(X_{ij}-X_{j}\right)/S_{j}$. *Z* is the standardized variable value, *X_ij_* is the original variable value, *X_j_* is the arithmetic average of the *j* index, and *S_j_* is the standard deviation of the *j* index.

In the process of Entropy analysis, the range method is used for data assimilation and dimensionless processing. The formula is as follows:

(4) $$X_{ij}^{\prime }=\frac{X_{ij}-\min(X_{1j},X_{2j},\cdots ,X_{nj})}{\max(X_{1j},X_{2j},\cdots ,X_{nj})-\min(X_{1j},X_{2j},\cdots ,X_{nj})}+1$$

(5) $$X_{ij}^{\prime }=\frac{\max(X_{1j},X_{2j},\cdots ,X_{nj})-X_{ij}}{\max(X_{1j},X_{2j},\cdots ,X_{nj})-\min(X_{1j},X_{2j},\cdots ,X_{nj})}+1$$

Among them, Formula (4) for the positive index operation and Formula (5) for the negative index operation.

2. Dimensionality Reduction Analysis of Principal Components

The principal component analysis of all the standardized index data is carried out by using the SPSS, and the eigenvalue and variance contribution rate is obtained. The eigenvector matrix of the principal component can be obtained by using the formula eigenvector value = component value/SQR (initial eigenvalue). Thus, the calculation formula of each principal component is as follows:

Where *F_ip_* is the score of the *p* principal component of the *i* year, *λ’_pj_* is the characteristic vector of the *j* index of the *p* principal component, and *ZX_ij_* is the standardized data of item *j* of the *i* year.

3. Entropy Method to Determine the Weight of Each Index

The entropy method determines the weight of each index layer according to the ordered degree of the information contained in each index. The greater the information entropy is, the smaller the index weight is; the smaller the information entropy is, the greater the index weight is. In the process of determining the index weight, the principal component analysis needs the variance contribution rate as the coefficient, including the subjective component, while the entropy method uses the information utility value to determine the index weight, which is an objective weighting method that can avoid the interference of human factors and make the evaluation results more objective.

Define the standardized formula as:

(6) $$f_{ij}=\frac{Y_{ij}}{\sum_{i=1}^{m}Y_{ij}}$$

Then the entropy value and information utility value of each principal component is calculated. The entropy value *e* of the index *j* is:

(7) $$e_{j}=-\frac{1}{\ln m}\sum_{i=1}^{m}f_{ij}\ln f_{ij}$$

The information utility value *d* of the index *j* is:

(8) $$d_{j}=1-e_{j}$$

In determining the entropy weight of each principal component, the greater the information utility value, the greater the entropy weight, indicating that the index is more important. The weight *W_j_* of the *j* indicator is:

(9) $$W_{j}=\frac{d_{j}}{\sum_{i=1}^{p}d_{j}}$$

4. Evaluation score of the entropy-principal component analysis

Through the analysis of the sample data, the principal component analysis is carried out by using SPSS software, and the principal component score is calculated; the entropy value of each principal component is calculated by using Excel, and the entropy weight of each principal component is obtained, thus Formula (12) is used to calculate the comprehensive score of the hazard index system of disaster-causing factors. The comprehensive score of the *i* sample is as follows:

(10) $$S_{i}=\sum_{j=1}^{m}W_{j}X_{ip}=W_{1}X_{i1}+W_{2}X_{i2}+\cdots +W_{j}X_{ip}$$

where *S_i_* is the comprehensive score of the *i* sample, and *X_ip_* is the score of the *p* principal component of the *i* sample. The lower the comprehensive score, the lower the risk of disaster factors, the smaller the risk of water resources carrying capacity.

## 3. Empirical Analysis and Discussion

### 3.1. Overview of Zhejiang Province

Zhejiang Province is located in the middle and lower reaches of the Yangtze River and borders Shanghai, Jiangsu, Anhui, Fujian, and other provinces. The average precipitation of the whole province in 2019 was 1949.9 mm, which was 18.9% more than that of the previous year and 21.6% more than that of many years. However, the temporal and spatial distribution of precipitation is uneven. The precipitation during the flood season (April to October) accounted for 69.0% of the whole year, generally showing a decreasing trend from west to east and from south to north, and the mountain area is larger than the plain. The coastal mountains are larger than the inland basins. In 2019, the per capita amount of water resources in Zhejiang Province was only 2280.8m^3^, which is low when compared globally.

### 3.2. The Vulnerability of the Disaster-Bearers

#### 3.2.1. Weight Calculation

The vulnerability index data of disaster victims are derived from the Statistical Yearbook of Zhejiang Province in 2020 and the Water Resources Bulletin of Zhejiang Province in 2019. In this paper, the weight of each index is calculated by the AHP method, as shown in Table 4.

According to Table 4, in the supporting force subsystem, the higher weight indicators are per capita water resources and per capita water supply, both of which are closely related to the total amount of local water resources, that is, the more abundant water resources in a region, the stronger its disaster supporting capacity. Zheng pointed out that water scarcity areas are more likely to face the risk of water resources overload, so it is difficult to provide a guarantee for coordinating the rational utilization of water resources [30].** However, in the regulating force subsystem, the development and utilization rate of water resources and ecological water use rate occupy a higher weight, that is, the higher the eco-environmental quality of an area, the stronger its ability to regulate and control the carrying risk of water resources. Song believed that for different types of water supply and ways of water use, ecological water demand should be ensured as a priority to meet the condition [31]. Planning water consumption quotas and increasing the repetition rate of industrial water use can effectively alleviate the pressure of water shortage.** In the pressure subsystem, because the risk pressure of water resources mainly comes from human economic and social activities, the weight gap between each index is not obvious. When studying the carrying capacity of water resources in Jiangsu Province, Li found that promoting water-saving activities and effective sewage discharge can effectively improve the carrying capacity of water resources [32].** At the same time, Tian pointed out that human protection of water resources and social and economic activities have an important impact on the carrying capacity of water resources. Tian also believed that banning sewage discharge and promoting a stricter water resources management system could effectively alleviate the pressure on water resources, thus reduce the cumulative risk of water resources [33].

#### 3.2.2. Calculation of Disaster Bearing Capacity and Construction of a Risk Matrix

According to Formulas (1) and (2), the vulnerability of disaster-bearers in Zhejiang Province is calculated and normalized.** The weights of each index of 4 and the results of each index of Table 4 are re-weighted and normalized, and the specific results are shown in Table 5. The results of calculation process are shown in Table A3 of Appendix A.

**Figure 2:**
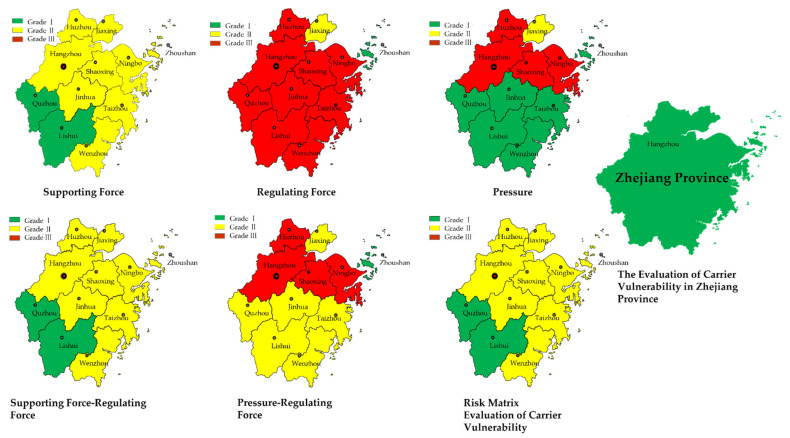
Evaluation grade of the risk matrix for the vulnerability of disaster-bearers in Zhejiang Province.

As shown by Table 4 and Table 5, because the supporting-regulating forces are positive indicators, the risk grade was determined by the highest value grade, while the supporting-pressure was a negative index, so the risk grade was determined by the lowest value level. For example, Hangzhou had the highest determined value of supporting force grade II, so its determined value is grade II, while the determined value of pressure grade III is the highest, so its fixed value is grade III, and so on. The supporting force, regulating force, and pressure risk grade of each city is obtained, and according to the composition rules of the Table 2 risk matrix, the vulnerability risk matrix of disaster-bearers in Zhejiang Province is obtained, and the specific results are shown in Figure 2.

From Figure 2, it can be seen that for supporting forces, except Quzhou and Lishui, the rest of the cities are grade II. This is because Quzhou and Lishui are located in the southwest of Zhejiang Province, with a large mountain forest area and a small population compared to other cities, so forest coverage, per capita water resources, and water supply will be at a higher level in the province.

As for the regulating force, Zhoushan is a grade I, Jiaxing grade II, and the others are grade III, indicating that the regulating force of most cities in Zhejiang Province is still at a low level. However, it is not difficult to find that the definite values of grade I and grade III in some of these cities are very close, so these cities can make continuous improvements to reduce the development and utilization rate of water resources and ecological water use rate. If we develop the economy on the basis of not destroying water resources, we can better improve the ability to bear and control disasters.** However, Quzhou and Lishui, which have excellent performance in supporting force, are in a backward stage, and even the grade I determination value is at a very low level, which may be due to the relatively backward economic development of the two places, and their poor performance in the province due to the low per capita GDP.

In terms of disaster-bearing pressure, it can be seen that southern Zhejiang is obviously better than northern Zhejiang, mainly because a large number of elements in northern Zhejiang continue to be concentrated in the region with the market-oriented reform, which stimulates the expansion of urban land. As a result, the urban development of northern Zhejiang is ahead of southern Zhejiang, highlighting the uneven development. However, the pressure of water resources in a region mainly comes from the population, ecological environment, technology, and economic level. Therefore, when urbanization is not as developed as northern Zhejiang, southern Zhejiang shows less pressure to bear disasters.** Hangzhou, Ningbo, Shaoxing, Jiaxing, Taizhou, and other cities are most closely related to the surrounding cities in the industrial economic network pattern. Hangzhou and Shaoxing play an important role in the intermediate transfer and guidance of the province’s industrial economy. As a result, these cities are facing greater pressure to bear disasters. However, in the Zhoushan archipelago, due to topography, natural conditions and other reasons, the level of urbanization is not at a high level, so its pressure is naturally small, and its determined value of less than 0.1 is at a higher level.** It is very interesting that although Quzhou and Lishui return to grade I again, and the level of industrialization is not high, most of them are extensive, but the gap between them is very obvious. This is because the distribution of industrial enterprises in Quzhou is greater than that in Lishui, for example, Zhejiang Juhua Group is located in Quzhou, while Lishui has few large industrial enterprises, and the government intends to abandon part of its economic development in Lishui to protect the ecological environment.

For the supporting-regulating force composite matrix, we can see that its grade is the same as the supporting force because they are both positive indicators, and there is no resistance to each other; thus, although the regulating force of each city is not good, because the supporting force is enough to cope with the risk of water resources carrying capacity, the primary idea is not to improve the ability to deal with risks by improving the regulation and control ability. However, for the pressure-regulating force, because the pressure is a negative index, there is a relationship between the two, so poor performance in any of them will have an impact on the overall grade. Although the pressure grade of Quzhou, Jinhua, Taizhou, Lishui, and Wenzhou is a grade I, because of their low regulating force, the grade of their composite matrix becomes grade II. This means that it is more likely to face the risk of carrying water resources.

For the final vulnerability of disaster-bearers risk matrix, because the dominance of the supporting-regulating force composite matrix is stronger than that of the pressure-regulating force composite matrix, the final grade of the risk matrix is consistent with the supporting-regulating force composite matrix. We can find that the supporting force plays an important role in the vulnerability of the disaster-bearers. Therefore, each region can give priority to ensuring that the supporting force is at a high grade, and for water-scarce areas, it is necessary to increase water saving and plant drought-tolerant trees so as to gradually increase the amount of water per capita in the future development. For non-water shortage areas, on the basis of water-saving concepts, the most important thing is to further improve the grade of supporting force by increasing forest coverage. Of course, the regulating force is not unimportant because, in most areas of Zhejiang, the subsystem is still at a low level, although it and the supporting force can jointly promote the prevention of water resources carrying risk; however, with the continuous increase in its grade, the pressure-regulating force can also be further enhanced so as to reduce the development pressure of the supporting force. Relatively speaking, the grade of supporting force of various cities in Zhejiang Province is relatively high, and the space for short-term progress is limited. However, there are still gaps in the regulating force that need to be continuously improved.

Finally, for Zhejiang as a whole, the risk matrix synthesis result is independent of the risk matrix synthesis result of each city. The results are a conclusion drawn by calculating the values of Zhejiang Province, using the same matrix judgment and synthesis method. It means that for Zhejiang, without subdividing into subordinate cities, the vulnerability of the disaster-bearers is a grade I, that is, it has a strong ability to cope with the risks carried by water resources. Zhejiang has strong economic strength and attaches great importance to economic development while protecting resources and the environment, paying attention to ecological development. Therefore, Zhejiang is not fragile in the face of water resources carrying risk.

### 3.3. Hazard of Disaster-Causing Factors

#### 3.3.1. Principal Component Analysis

The index data of hazards of disaster-causing factors are derived from the Statistical Yearbook of Zhejiang Province in 2020 and the Water Resources Bulletin of Zhejiang Province in 2019. According to Formulas (3) and (6), this paper uses SPSS software to analyze the principal components of the data and the results are shown in Table A4 and Table A5 of Appendix A. The specific results show that there are five principal components with eigenvalues greater than 1, and the cumulative variance contribution of the first four principal components is 84.955%, close to 85%. Therefore, we can determine that the first four principal components have a great impact on the risk of disaster-causing factors in Zhejiang Province, which is consistent with the number of Table 3 subsystems. There are also more indicators with higher scores of the first principal component, but most of them are concentrated in the industrial structure subsystem, so we determine that the first principal component is divided into the industrial structure subsystem. By analogy, it was concluded that the second principal component is the climate change subsystem; the third principal component is the water use efficiency subsystem, and; the fourth principal component is the population structure subsystem.

#### 3.3.2. Entropy Weight Calculation

According to Formulas (3)–(5) and (7)–(10), the risk entropy, information utility, and weight of disaster-causing factors in Zhejiang Province are calculated, and the specific results are shown in Table 6.

From Table 6, we can know that the *e_j_* value of each index is more than 0.9 because the greater the entropy value, the smaller the amount of information and the worse the stability of the system is. Therefore, it can be observed that the degree of disorder of each index is at a high level, indicating that the study of the risk of disaster factors is of significance. Although the overall risk of disaster-causing factors is at a disordered level, there is still a relative gap in the *d_j_* of each index, and the larger the *d_j_*, the greater the impact on the evaluation; thus, the *W_j_* of each index is also different.

#### 3.3.3. Comprehensive Score Calculation of Entropy-Principal Component Analysis

According to Formula (11), and combined with the specific data of Table 6, the comprehensive risk score of disaster-causing factors in Zhejiang Province was calculated and ranked, and the specific results are shown in Table 7.

For the table showing the risk scores of disaster-causing factors, the smaller the score, the smaller the risk, and the higher the ranking. Therefore, from Table 7, it can be seen that the risk of disaster factors in southern Zhejiang is at a greater risk than that in northern Zhejiang and that in western Zhejiang is at a greater risk than that in eastern Zhejiang, but the overall score gap is not large, which is consistent with the results of Shen through the study of water security status and its spatio-temporal variation characteristics in Zhejiang Province. Shen believes that from the spatial level, the spatial heterogeneity of water security in Zhejiang Province is significant, showing the characteristics of “strong in the northeast and weak in the southwest”, which is consistent with the pattern of economic and social development of the province; from the level of dynamic change, the regional gap of water security in Zhejiang Province is gradually narrowing, and its optimization speed shows a pattern of “slow in the northeast and fast in the southeast” [34].

As for the industrial structure of the first principal component, the economic level and industrial layout of Zhejiang are stronger in the northeast than in the southwest, and the development level of intelligent industries, green economy, and other emerging fields in Zhejiang cities is also uneven. Additionally, digital economy plays an increasingly important role in the development of China, and it also plays an important role in the field of water resources, such as intelligent water conservancy, intelligent water affairs, intelligent water control, and other related research. Therefore, cities such as Hangzhou, which are leading in the development of the digital economy, have an advantage in this respect.

For the second principal component of climate change, it is not difficult to find that Zhejiang Province has a small area, subtropical monsoon climate, plum rainy season, typhoon season, and other periods, so its precipitation resources are abundant and the climate is appropriate. As a result, the difference between cities is not obvious. Therefore, actively dealing with climate change and preventing global warming can effectively reduce the risk of disaster factors.

As for the water use efficiency of the third principal component, because the main body of water use is small but dense, and the water use behavior is complex, it can be found that the water in the water use efficiency mainly comes from the second principal component and is used in the first principal component, which is dominated by the other two. Therefore, water use efficiency ranks as the third principal component. Extensive and intense human activities will cause serious water environment pollution, which leads to the deterioration of the use of water resources, which not only harms water resources but also becomes a major bottleneck restricting the sustainable development of human society. In 2013, China issued the strictest Water Resources Management system, and at the end of the same year, Zhejiang Province put forward “five-water co-governance”, which earlier regulated the water use behavior and effectively improved the water use efficiency; thus, it can be found that the overall performance of Zhejiang Province is better; and there is little difference between cities.

As for the population structure of the fourth principal component, it can be found that the proportion of population density and urban built-up area is relatively high, mainly because the area of Zhejiang Province is small and the level of economic development is high. The continuous increase in the population has led to greater population pressure in limited areas and the expansion of urban areas, thus affecting the ecological environment in many ways, resulting in an increased risk of disaster-causing factors. The disorderly expansion of urban space leads to the destruction of the water circulation system, and the excessive growth of population and the serious lag in the construction of the water supply network and sewage treatment system seriously affect the degree of coordination. Wang also pointed out that measures such as reducing the population growth rate, improving the water use efficiency of the economic system, and optimizing the allocation of water conservancy facilities can effectively improve the carrying capacity of water resources [35]. Therefore, Zhejiang Province should not “change” the economy with people and cities in the process of development, but should comprehensively consider the coordinated development of population, economy, water resources, and other factors so as to reduce the harm of water resources that may be brought in the process of economic construction.

### 3.4. Risk Assessment of Water Resources Carrying Capacity

**Figure 3:**
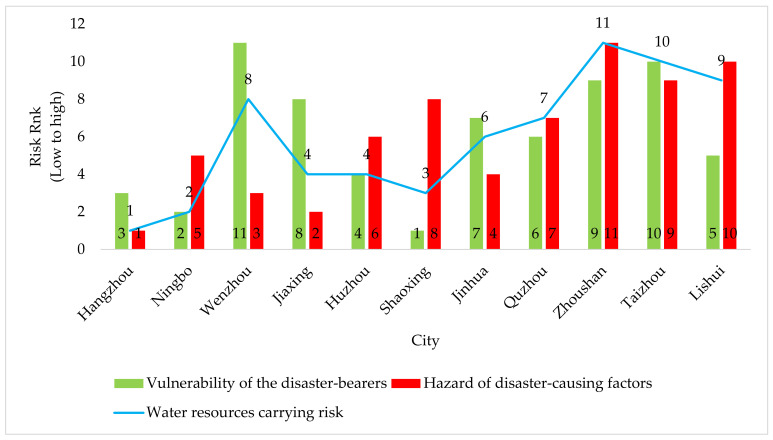
Each city’s ranking of vulnerability, hazards, and carrying risks of water resources in Zhejiang Province.

The operational Formulas (3)–(5) and (7)–(10) were used to calculate the entropy weight of each subsystem of the vulnerability of disaster-bearers in Zhejiang Province and to rank each subsystem in Table 5 after weighted addition and re-rank the risk of water resources carrying capacity of cities in Zhejiang Province after adding it with the risk ranking of disaster-causing factors in Table 7. The grades from 1 to 11 mean that the vulnerability of the disaster-bearers, the risk of disaster-causing factors, and the carrying risk of water resources all change from small to large, as shown by Figure 3.

As can be seen from Figure 3, the risk of water resources carrying capacity in economically developed cities such as Hangzhou, Ningbo, and Shaoxing is relatively small, while the relatively less developed cities such as Zhoushan, Lishui, and Quzhou are faced with greater risk of water resources carrying capacity. However, the economic level is not the main factor, such as Wenzhou and Taizhou, their economic level does not belong to the backward position in Zhejiang, but the risk to carrying water resources is still large because they are located in coastal areas and are large cities affected by typhoons. Typhoon transit will have a greater impact on the carrying capacity of local water resources.** Typhoons not only cause serious rain and waterlogging, but also have a great impact on economic and social operations. Therefore, the risk to water resources carrying in Wenzhou and Taizhou is not only affected by human activities but also by typhoon-related factors. As far as Wenzhou is concerned, the overall level of Wenzhou is not high because its vulnerability ranks last, which indicates that Wenzhou needs to strengthen the construction of bearing capacity and pay attention to the influence of the risk of disaster-causing factors. As far as Taizhou is concerned, the vulnerability of disaster-bearers and the risk of disaster-causing factors are poor, and the degree of risk to water resources carrying capacity of Taizhou may be higher than that of Zhoushan under the influence of typhoon factors; thus, whether by the water-saving behavior in daily economic activities or the protective behavior in response to typhoons, Taizhou urgently needs to be strengthened, and there is a lot of room for improvement.

For Hangzhou, Ningbo, Shaoxing, and other places, as the urban economic development is relatively high, and the level of investment in water resources management and protection has been in the leading position in the province, which needs to be maintained. One should pay attention to the prevention of non-procedural water resources carrying risks. For Quzhou and Lishui, it can be observed that the vulnerability of the disaster-bearers is in the middle level, but the risk of disaster-causing factors is lower. This is mainly because the eco-environmental level of Quzhou and Lishui is high within the province, which can slightly make up for the deficiency of their vulnerability due to lack of economic development; however, this is not enough to significantly reduce the possibility of water resources carrying risk, resulting in the risk of disaster-causing factors still at a high level, which affects the risk level of water resources carrying capacity.

Finally, as a special island city, Zhoushan Archipelago is affected by many factors, such as geographical environment, economic development, and natural resources. Due to the different geographical characteristics of the islands, there are obvious differences in their industrial structure and the tolerance and coping ability of people in response to natural disasters; the Zhoushan Archipelago area has been prone to storm surges, typhoons, water and other natural disasters since ancient times, so the impact of these on the economic and social life of the island area cannot be ignored. Therefore, in the future, Zhoushan Islands should reasonably control the population, promote technological innovation, improve the efficiency of resource and energy utilization, actively develop the environmental protection industry, and reduce the demand for natural capital. We suggest promoting the coordinated development of human society and the ecosystem so as to reduce the risk of water resources carrying capacity.

## 4. Deficiency

As a new research field in China, there are still many imperfections in water resources carrying risk assessments, so there are some deficiencies in this study. First of all, this paper directly takes the index system of water resources carrying capacity as the index system of water resources carrying vulnerability, and there may be some indicators that cannot fully explain the concept of “vulnerability”, which leads to some deviation in the research. Secondly, the risk matrix grading rules only follow the practice of predecessors and do not change according to the specific research, which may also make the final grading of some cities biased against the real situation. Finally, in the study of the risk of disaster-causing factors, this paper finds that the geographical situation and typhoon climate have an impact on the carrying risk of water resources in an area, but it has not been studied in this paper. Additionally, the specific behavior changes of the government and the public in the face of water resources carrying risk had not been studied in this paper. Therefore, these can be used for future research to continuously improve the risk of water resources carrying capacity.

## 5. Conclusions

This paper studies the carrying risk of water resources in Zhejiang Province from two aspects: the vulnerability of disaster-bearers and the risk of disaster-causing factors. The Analytic Hierarchy Process-Fuzzy Comprehensive Evaluation method and Entropy-Principal Component Analysis method are used, respectively, for the two aspects, and a risk matrix was constructed for the vulnerability of disaster-bearers. Finally, the ranking and specific conditions of water resources carrying risks of various cities in Zhejiang Province are listed by combining the evaluation results of the two aspects, and the conclusions are as follows.

Zhejiang Province has a strong ability to cope with the water resource carrying risk, but there are still deficiencies in some cities. For the vulnerability of the disaster-bearers, it shows that the northeast region is more vulnerable than the southwest region, and the role of supporting force is more obvious. As for the risk of disaster-causing factors, industrial structure, climate change, water resources utilization efficiency, and population structure have a great impact. The risk of disaster factors in southern Zhejiang is at great risk than that in the north, and western Zhejiang is at a greater risk than that in the east. Generally speaking, Zhejiang Province shows a low risk of carrying water resources in areas with a higher economic level. Therefore, cities in Zhejiang Province can promote energy conservation and emission reduction, encourage water-saving behavior, improve water use efficiency, promote the coordinated development of economy, society, and ecological environment, and reduce the risk of carrying water resources by optimizing the industrial layout and population structure.

To conclude, the risk of carrying capacity of water resources is affected by many aspects, and all of them should be taken into account when preventing the risk. For the government, the most important thing is to measure whether the speed of economic and social development is in line with the state of water resources, whether the development of the economy has caused damage to water resources at the same time; whether water resources are reasonably developed and utilized, and its effective recycling is promoted, and; the government can effectively restrict the behavior of enterprises and the public by promulgating legal provisions. For the public, whether they have water-saving awareness, whether to maintain good daily water-saving behavior and actively participate in water-saving activities are conducive to reducing the risk of water resources carrying capacity.

## Figures and Tables

**Table 1:** Index system and grade classification standard of the vulnerability of disaster-bearers.

First-Level Index	Second-Level Index	Type	Grading Standard		
			Grade I (Not Vulnerable)	Grade II (A Little Vulnerable)	Grade III (Vulnerable)
Supporting force	*C_1_* Per capita water resources (m^3^)	P	>1670	1000~1670	<1000
	*C_2_* Water production modulus (10^4^ m^3^/km^2^)	P	>80	50~80	<50
	*C_3_* Per capita water supply (m^3^)	P	>450	350~450	<350
	*C_4_* Forest coverage (%)	P	>40	25~40	<25
Regulating force	*C_5_* Development and utilization of water resources(%)	P	<40	40~70	>70
	*C_6_* Per capita GDP(Yuan)	P	>24840	6624~24840	<6624
	*C_7_* Ecological water consumption rate (%)	P	>5	1~5	<1
Pressure	*C_8_* Per capita domestic water consumption(L)	N	<70	70~180	>180
	*C_9_* Water consumption of GDP (m^3^/10^4^ Yuan)	N	<100	100~400	>400
	*C_10_* Water consumption of industrial added value (m^3^/10^4^ Yuan)	N	<50	50~200	>200
	*C_11_* Population density (Person/km^2^)	N	<200	200~500	>500
	*C_12_* Urbanization rate (%)	N	<50	50~80	>80
	*C_13_* Farmland irrigation quota (m^3^/km^2^)	N	<250	250~400	>400

P means positive, N means negative. The calculating methods of each secondary index are shown in Table A1 of Appendix A.

**Table 2:** Vulnerability risk matrix of disaster-bearers.

Regulating Force	Supporting Force			Regulating Force	Pressure			Pressure-Regulating	Supporting-Regulating		
	Grade I	Grade II	Grade III		Grade I	Grade II	Grade III		Grade I	Grade II	Grade III
Grade I	I	II	II	Grade I	I	II	II	Grade I	I	II	II
Grade II	I	II	III	Grade II	I	II	II	Grade II	I	II	III
Grade III	I	II	III	Grade III	I	II	III	Grade III	II	II	III

The first four columns are disaster-bearers supporting-regulating force risk matrix; the middle four columns are disaster-bearers pressure-regulating force risk matrix; the last four columns are disaster-bearers supporting-regulating force and pressure-regulating force risk matrix.

**Table 3:** Index system of hazards of disaster-causing factors.

First-Level Index	Second-Level Index	Type
Climate change	*X*_11_ Average air temperature (°C)	N
	*X*_12_ Average precipitation (mm)	P
	*X*_13_ Surface moisture index	P
	*X*_14_ Surface drought index	N
	*X*_15_ Total amount of water resources (10^8^m^3^)	P
Population structure	*X*_21_ Population density (person/km^2^)	N
	*X*_22_ Urbanization rate (%)	N
	*X*_23_ Registered population (10^4^ person)	N
	*X*_24_ Natural population growth rate (%)	N
	*X*_25_ Proportion of urban built-up area (%)	N
Industrial structure	*X*_31_GDP(10^8^ Yuan)	N
	*X*_32_ GDP per capita (10^4^ Yuan/person)	N
	*X*_33_ The growth rate of GDP(%)	N
	*X*_34_ Proportion of tertiary industry (%)	N
	*X*_35_ Proportion of primary industry (%)	N
Water use efficiency	*X*_41_ Industrial water quota (10^4^ m^3^/day)	N
	*X*_42_ Water consumption of GDP (m^3^/10^4^ Yuan)	N
	*X*_43_** Farmland irrigation quota(m^3^/mu)	N
	*X*_44_ Development and utilization of water resources (%)	N
	*X*_45_ Domestic water quota (10^4^ m^3^/day)	N

P means positive, N means negative. The calculating methods of each secondary index are shown in Table A2 of Appendix A.

**Table 4:** Weight table of vulnerability indicators for the vulnerability of disaster-bearers.

First-Level Index	Supporting Force				Regulating Force			Pressure					
Second-level index	*C_1_*	*C_2_*	*C_3_*	*C_4_*	*C_5_*	*C_6_*	*C_7_*	*C_8_*	*C_9_*	*C_10_*	*C_11_*	*C_12_*	*C_13_*
Weight	0.36	0.21	0.28	0.15	0.43	0.24	0.33	0.26	0.22	0.11	0.17	0.10	0.15

**Table 5:** Normalized results of measured values of each subsystem.

City	Supporting Force			Regulating Force			Pressure		
	Grade I	Grade II	Grade III	Grade I	Grade II	Grade III	Grade I	Grade II	Grade III
Hangzhou	0.39	0.45	0.16	0.24	0.35	0.42	0.40	0.33	0.27
Ningbo	0.28	0.48	0.24	0.37	0.24	0.40	0.40	0.32	0.28
Wenzhou	0.30	0.57	0.13	0.28	0.16	0.56	0.14	0.16	0.71
Jiaxing	0.00	0.56	0.44	0.29	0.38	0.33	0.04	0.84	0.12
Huzhou	0.39	0.45	0.16	0.38	0.22	0.40	0.44	0.17	0.39
Shaoxing	0.41	0.43	0.16	0.30	0.27	0.44	0.37	0.31	0.33
Jinhua	0.36	0.49	0.15	0.17	0.19	0.64	0.19	0.27	0.54
Quzhou	0.51	0.34	0.15	0.12	0.31	0.58	0.33	0.24	0.43
Zhoushan	0.12	0.60	0.29	0.41	0.18	0.41	0.08	0.21	0.72
Taizhou	0.36	0.52	0.12	0.18	0.17	0.66	0.16	0.22	0.62
Lishui	0.41	0.36	0.23	0.11	0.28	0.61	0.19	0.22	0.59
Zhejiang	0.43	0.43	0.14	0.16	0.33	0.51	0.32	0.28	0.41

**Table 6:** Entropy value and weight of each index.

**Index**	***X*_11_**	***X*_12_**	***X*_13_**	***X*_14_**	***X*_15_**	***X*_21_**	***X*_22_**	***X*_23_**	***X*_24_**	***X*_25_**
*e_j_*	0.988	0.993	0.992	0.995	0.992	0.994	0.994	0.991	0.989	0.993
*d_j_*	0.012	0.007	0.008	0.005	0.008	0.006	0.006	0.009	0.011	0.007
*W_j_*	7.98%	4.52%	5.05%	3.42%	5.30%	4.23%	4.10%	5.70%	7.33%	4.60%
**Index**	***X*_31_**	***X*_32_**	***X*_33_**	***X*_34_**	***X*_35_**	***X*_41_**	***X*_42_**	***X*_43_**	***X*_44_**	***X*_45_**
*e_j_*	0.993	0.991	0.991	0.994	0.994	0.993	0.994	0.991	0.995	0.991
*d_j_*	0.007	0.009	0.009	0.006	0.006	0.007	0.006	0.009	0.005	0.009
*W_j_*	4.69%	6.00%	6.00%	3.96%	4.14%	4.42%	3.61%	5.79%	3.32%	5.82%

**Table 7:** Comprehensive evaluation value of hazard of disaster-causing factors in Zhejiang Province. (×10^−4^).

**City/Index**	***X*_11_**	***X*_12_**	***X*_13_**	***X*_14_**	***X*_15_**	***X*_21_**	***X*_22_**	***X*_23_**	***X*_24_**	***X*_25_**	***X*_31_**
Hangzhou	6.13	0.09	2.00	−1.97	5.53	1.60	0.04	0.59	2.08	0.86	2.18
Ningbo	6.72	0.11	2.01	−1.97	4.74	1.34	0.05	0.74	2.63	1.03	2.71
Wenzhou	3.56	0.09	2.12	−2.01	4.93	1.44	0.06	0.57	2.25	0.95	3.54
Jiaxing	7.12	0.08	1.77	−1.04	3.34	1.06	0.07	0.93	2.16	0.62	3.74
Huzhou	7.12	0.07	1.9	−1.88	3.61	1.70	0.07	1.00	3.18	1.13	4.09
Shaoxing	5.54	0.09	1.95	−1.93	4.18	1.61	0.07	0.86	3.56	0.89	3.67
Jinhua	4.35	0.10	2.16	−2.02	4.86	1.71	0.06	0.83	1.78	1.14	3.86
Quzhou	5.14	0.12	2.33	−2.04	4.81	1.98	0.08	1.01	2.59	0.84	4.33
Zhoushan	7.12	0.13	2.22	−2.03	3.09	1.40	0.07	1.13	3.48	1.25	4.36
Taizhou	4.75	0.11	2.23	−2.03	4.99	1.61	0.08	0.74	2.16	1.13	3.77
Lishui	3.56	0.10	3.55	−2.08	6.18	2.11	0.08	1.00	1.91	1.19	4.34
**City/Index**	***X*_32_**	***X*_33_**	***X*_34_**	***X*_35_**	***X*_41_**	***X*_42_**	***X*_43_**	***X*_44_**	***X*_45_**	**Score**	**Rank**
Hangzhou	2.40	2.78	0.14	−3.43	−0.01	2.85	3.15	1.20	−0.73	27.47	1
Ningbo	2.66	3.32	0.25	−3.32	−0.02	2.93	4.85	1.21	−0.82	31.16	5
Wenzhou	4.69	3.68	0.21	−3.40	−0.02	2.70	3.94	1.26	−0.93	29.63	3
Jiaxing	3.50	3.63	0.28	−3.41	−0.02	2.50	3.13	0.69	−1.2	28.95	2
Huzhou	3.79	2.53	0.28	−3.00	−0.03	2.31	3.93	1.09	−1.32	31.55	6
Shaoxing	3.45	4.46	0.25	−3.13	−0.02	2.58	4.32	1.16	−1.15	32.39	8
Jinhua	4.41	3.39	0.20	−3.22	−0.02	2.47	4.87	1.27	−1.18	31.03	4
Quzhou	4.69	4.39	0.22	−2.75	−0.02	1.54	3.24	1.31	−1.42	32.38	7
Zhoushan	3.45	5.06	0.21	−1.72	−0.03	3.07	6.26	1.27	−1.47	38.33	11
Taizhou	4.32	4.79	0.25	−2.76	−0.02	2.63	3.92	1.29	−1.15	32.81	9
Lishui	4.80	4.66	0.21	−2.50	−0.03	2.23	4.16	1.38	−1.42	35.43	10

## Data Availability

The data of this paper are derived from the Statistical Yearbook of Zhejiang Province in 2020 and the Water Resources Bulletin of Zhejiang Province in 2019. The link to the Statistical Yearbook of Zhejiang Province in 2020 is http://tjj.zj.gov.cn/flash/tjj/Reports1/2020-%E7%BB%9F%E8%AE%A1%E5%B9%B4%E9%89%B40115/indexcn.html (accessed on 15 April 2021). The link to the Water Resources Bulletin of Zhejiang Province in 2019 is http://www.zjsw.cn/pages/doc.jsp?docId=1658196&catId=1029 (accessed on 15 April 2021). If you are interested in this study and want to obtain the original data, you can contact us.

## Appendix A

This is the calculation method of each secondary index in Table 1 of Section 2.1.1, as shown in Table A1.

**Table A1:** Calculation method of each secondary index in Table 1.

First-Level Index	Second-Level Index	Calculating Method
Supporting force	*C_1_*	Total water resources/Total resident population
	*C_2_*	Total water resources/Regional area
	*C_3_*	Total annual water supply/Total resident population
	*C_4_*	Forest area/Total land area
Regulating force	*C_5_*	Total water supply/Total water resources
	*C_6_*	Total value of GDP/Total population
	*C_7_*	Eco-environmental water consumption/Total water consumption
Pressure	*C_8_*	Total daily water consumption/Total resident population
	*C_9_*	Total socio-economic water consumption/GDP
	*C_10_*	Industrial water consumption/Industrial value added
	*C_11_*	Resident population/Regional area
	*C_12_*	Urban population/total population
	*C_13_*	Irrigation water consumption/Irrigation area

This is the calculation method of each secondary index in Table 3 of Section 2.2.1, as shown in Table A2.

**Table A2:** Calculation method of each secondary index in Table 3.

First-Level Index	Second-Level Index	Calculating Method
Climate change	*X*_11_	Σ** Monthly average temperature/12
	*X*_12_	Σ Monthly average precipitation
	*X*_13_	Land income moisture/Ground expenditure water
	*X*_14_	Ground expenditure water/Land income moisture
	*X*_15_	Total amount of water resources
Population structure	*X*_21_	Resident population/Regional area
	*X*_22_	Urban population/Total population
	*X*_23_	Total resident population
	*X*_24_	Natural population growth rate
	*X*_25_	Urban area/Regional area
Industrial structure	*X*_31_	GDP
	*X*_32_	GDP/Total population
	*X*_33_	(GDP in this yeay/GDP in last year)-1
	*X*_34_	Output value of tertiary industry/Total output value
	*X*_35_	Output value of primary industry/Total output value
Water use efficiency	*X*_41_	Total industrial water consumption/365
	*X*_42_	Total social and economic water consumption/GDP
	*X*_43_	Total amount of irrigation water for farmland/Farmland area
	*X*_44_	Regional water consumption/Total amount of water resources
	*X*_45_	Total domestic water consumption/365

These are data during the calculation process of Table 5 of Section 3.2.2, as shown in Table A3.

**Table A3:** The results of various indexes shows the vulnerability of disaster-bearers in Zhejiang Province.

Evaluation Index	Zhejiang Province			City														
				Hangzhou			Ningbo			Wenzhou			Jiaxing			Huzhou		
	*Uv_1_*	*Uv_2_*	*Uv_3_*	*Uv_1_*	*Uv_2_*	*Uv_3_*	*Uv_1_*	*Uv_2_*	*Uv_3_*	*Uv_1_*	*Uv_2_*	*Uv_3_*	*Uv_1_*	*Uv_2_*	*Uv_3_*	*Uv_1_*	*Uv_2_*	*Uv_3_*
*C1*	0.43	0.05	0.53	0.28	0.15	0.57	0.11	0.25	0.64	0.13	0.24	0.63	0.00	1.00	0.00	0.22	0.19	0.59
*C2*	0.25	0.17	0.58	0.28	0.15	0.58	0.22	0.19	0.59	0.38	0.08	0.54	0.00	1.00	0.00	0.12	0.18	0.71
*C3*	0.00	1.00	0.00	0.00	1.00	0.00	0.00	1.00	0.00	0.00	1.00	0.00	0.00	0.72	0.28	0.04	0.23	0.73
*C4*	0.40	0.07	0.54	0.42	0.05	0.53	0.34	0.11	0.55	0.41	0.06	0.53	0.00	0.51	0.49	0.34	0.10	0.55
*C5*	0.37	0.09	0.55	0.37	0.09	0.54	0.37	0.09	0.54	0.38	0.08	0.54	0.01	0.12	0.88	0.34	0.11	0.56
*C6*	0.46	0.03	0.51	0.48	0.02	0.51	0.47	0.02	0.51	0.44	0.04	0.52	0.47	0.02	0.51	0.46	0.03	0.51
*C7*	0.00	1.00	0.00	0.00	0.98	0.02	0.00	0.54	0.46	0.33	0.12	0.56	0.00	0.60	0.40	0.00	0.74	0.26
*C8*	0.00	0.96	0.04	0.00	0.80	0.21	0.00	0.68	0.32	0.00	0.05	0.95	0.00	0.92	0.08	0.00	0.88	0.12
*C9*	0.28	0.15	0.57	0.29	0.14	0.57	0.29	0.14	0.57	0.29	0.14	0.57	0.28	0.15	0.57	0.27	0.15	0.58
*C10*	0.08	0.25	0.67	0.20	0.20	0.60	0.24	0.17	0.59	0.12	0.24	0.64	0.20	0.20	0.60	0.21	0.19	0.60
*C11*	0.00	1.00	0.00	0.00	1.00	0.00	0.00	1.00	0.00	0.00	1.00	0.00	0.00	1.00	0.00	0.00	1.00	0.00
*C12*	0.00	0.90	0.10	0.00	0.55	0.45	0.00	0.71	0.29	0.00	0.82	0.18	0.00	0.92	0.08	0.00	0.06	0.94
*C13*	0.02	0.19	0.79	0.00	0.50	0.50	0.19	0.20	0.60	0.00	0.96	0.04	0.00	1.00	0.00	0.00	0.95	0.05
**Evaluation index**	**Shaoxing**			**Jinhua**			**Quzhou**			**Zhoushan**			**Taizhou**			**Lishui**		
*C1*	0.28	0.14	0.57	0.40	0.07	0.53	0.48	0.02	0.51	0.00	0.92	0.08	0.39	0.07	0.54	0.49	0.01	0.50
*C2*	0.28	0.20	0.52	0.25	0.17	0.58	0.39	0.07	0.54	0.00	0.65	0.35	0.31	0.13	0.56	0.38	0.08	0.54
*C3*	0.00	1.00	0.00	0.00	1.00	0.00	0.32	0.12	0.56	0.00	1.00	0.00	0.00	1.00	0.00	0.00	1.00	0.00
*C4*	0.39	0.08	0.54	0.41	0.06	0.53	0.43	0.04	0.52	0.36	0.10	0.55	0.41	0.06	0.53	0.45	0.04	0.52
*C5*	0.36	0.09	0.55	0.38	0.08	0.54	0.39	0.07	0.54	0.38	0.08	0.54	0.39	0.08	0.54	0.40	0.07	0.53
*C6*	0.47	0.02	0.51	0.45	0.03	0.52	0.44	0.04	0.52	0.47	0.02	0.51	0.45	0.03	0.52	0.44	0.04	0.52
*C7*	0.00	0.81	0.20	0.24	0.17	0.59	0.00	0.75	0.25	0.00	1.00	0.00	0.33	0.12	0.56	0.00	0.64	0.36
*C8*	0.00	0.81	0.19	0.00	0.11	0.88	0.07	0.25	0.68	0.00	0.11	0.89	0.00	0.11	0.89	0.05	0.24	0.71
*C9*	0.28	0.15	0.57	0.28	0.15	0.57	0.24	0.17	0.59	0.30	0.14	0.57	0.28	0.15	0.57	0.27	0.15	0.58
*C10*	0.16	0.23	0.62	0.00	0.81	0.19	0.00	0.67	0.33	0.00	0.60	0.40	0.14	0.24	0.63	0.16	0.23	0.62
*C11*	0.00	1.00	0.00	0.00	1.00	0.00	0.12	0.24	0.64	0.00	1.00	0.00	0.00	1.00	0.00	0.28	0.15	0.57
*C12*	0.00	0.89	0.11	0.00	0.88	0.12	0.05	0.24	0.71	0.00	0.88	0.12	0.01	0.12	0.87	0.01	0.16	0.83
*C13*	0.05	0.24	0.71	0.14	0.43	0.43	0.00	0.55	0.45	0.37	0.09	0.54	0.00	0.95	0.05	0.02	0.19	0.80

These are the specific results of the principal component analysis in Section 3.3.1, as shown in Table A4 and Table A5.

**Table A4:** Characteristic Root and Cumulative contribution rate of correlation Matrix.

Principal Component	Before Rotation			After Rotation		
	Total	Variance %	Accumulation %	Total	Variance %	Accumulation %
1	7.525	37.627	37.627	6.212	31.060	31.060
2	4.792	23.962	61.589	4.044	20.221	51.280
3	3.486	17.429	79.018	2.932	14.662	65.942
4	1.187	5.937	84.955	2.533	12.663	78.605
5	1.030	5.152	90.107	2.300	11.502	90.107

**Table A5:** Component score coefficient matrix.

Subsystems	Standardized Index	Component Score			
		1	2	3	4
Climate change	Average air temperature	−0.374	0.766	0.258	0.247
	Average precipitation	−0.513	0.024	−0.496	0.412
	Surface moisture index	−0.695	0.480	0.077	0.082
	Surface drought index	0.458	−0.613	0.470	0.329
	Total amount of water resources	−0.079	0.949	0.133	0.019
Population structure	Population density	0.643	−0.555	−0.217	0.415
	Urbanization rate	0.774	0.214	−0.539	0.018
	Registered population	0.678	0.609	−0.051	0.165
	Natural population growth rate	0.156	0.637	0.392	0.379
	Proportion of urban built-up area	0.613	−0.164	0.546	0.240
Industrial structure	GDP	0.865	0.355	−0.280	−0.034
	GDP per capita	0.702	−0.242	−0.502	−0.170
	The growth rate of GDP	0.712	0.094	0.152	−0.571
	Proportion of tertiary industry	0.063	0.703	−0.367	0.029
	Proportion of primary industry	−0.788	−0.267	−0.475	0.060
Water use efficiency	Industrial water quota	0.883	0.319	−0.006	0.010
	Water consumption of GDP	−0.427	0.099	0.770	−0.232
	Farmland irrigation quota	0.399	0.229	0.790	−0.042
	Development and utilization of water resources	0.575	−0.665	0.395	0.149
	Domestic water quota	0.853	0.421	−0.220	0.044
